# Design of Tree-Frog-Inspired Adhesives

**DOI:** 10.1093/icb/icaa037

**Published:** 2020-05-15

**Authors:** Julian K A Langowski, Dimitra Dodou, Peter van Assenbergh, Johan L van Leeuwen

**Affiliations:** Experimental Zoology Group, Wageningen University & Research, De Elst 1, Wageningen, 6708 WD, The Netherlands; Department of BioMechanical Engineering, Delft University of Technology, Mekelweg 2, Delft, 2628 CD, The Netherlands; Department of BioMechanical Engineering, Delft University of Technology, Mekelweg 2, Delft, 2628 CD, The Netherlands; Experimental Zoology Group, Wageningen University & Research, De Elst 1, Wageningen, 6708 WD, The Netherlands

## Abstract

The adhesive toe pads of tree frogs have inspired the design of various so-called ‘smooth’ synthetic adhesives for wet environments. However, these adhesives do not reach the attachment performance of their biological models in terms of contact formation, maintenance of attachment, and detachment. In tree frogs, attachment is facilitated by an interconnected ensemble of superficial and internal morphological components, which together form a functional unit. To help bridging the gap between biological and bioinspired adhesives, in this review, we (1) provide an overview of the functional components of tree frog toe pads, (2) investigate which of these components (and attachment mechanisms implemented therein) have already been transferred into synthetic adhesives, and (3) highlight functional analogies between existing synthetic adhesives and tree frogs regarding the fundamental mechanisms of attachment. We found that most existing tree-frog-inspired adhesives mimic the micropatterned surface of the ventral epidermis of frog pads. Geometrical and material properties differ between these synthetic adhesives and their biological model, which indicates similarity in appearance rather than function. Important internal functional components such as fiber-reinforcement and muscle fibers for attachment control have not been considered in the design of tree-frog-inspired adhesives. Experimental work on tree-frog-inspired adhesives suggests that the micropatterning of adhesives with low-aspect-ratio pillars enables crack arresting and the drainage of interstitial liquids, which both facilitate the generation of van der Waals forces. Our analysis of experimental work on tree-frog-inspired adhesives indicates that interstitial liquids such as the mucus secreted by tree frogs play a role in detachment. Based on these findings, we provide suggestions for the future design of biomimetic adhesives. Specifically, we propose to implement internal fiber-reinforcements inspired by the fibrous structures in frog pads to create mechanically reinforced soft adhesives for high-load applications. Contractile components may stimulate the design of actuated synthetic adhesives with fine-tunable control of attachment strength. An integrative approach is needed for the design of tree-frog-inspired adhesives that are functionally analogous with their biological paradigm.

## Introduction

Bioadhesion is an interdisciplinary research field at the interface of biology, physics, and chemistry, which stimulates research on the fundamentals of adhesion and friction (Jagota and Hui 2011; Federle and Labonte 2019), provides insights into the evolution of biological adhesive systems (Büscher et al. 2018; Gamel et al. 2019; Russell and Gamble 2019), and generates inspiration for the design of synthetic micropatterned adhesive surfaces (henceforth referred to as ‘adhesives’; Li et al. 2016; Eisenhaure and Kim 2017). Numerous bioinspired adhesives have been developed in the past two decades. These adhesives typically are classified into ‘hairy’ (i.e., fibrillar) adhesives, inspired by the dry adhesive pads of geckos and other animals possessing high-aspect-ratio hair-like structures (Autumn et al. 2002; Federle 2006), and ‘smooth’ adhesives, inspired by the adhesive pads of tree frogs and other animals bearing low-aspect-ratio pillar-like structures (Hanna and Barnes 1991; Gorb et al. 2000).

The transfer of functional principles from the biological to the technological domain is a central concept in the design of bioinspired adhesives (Biomimetics 2012). The degree of analogy resulting from this transfer can be expressed in terms of functionality, or structure and material (Farzaneh and Lindemann 2019). Functionally analogous (i.e., biomimetic) adhesives function in a similar manner as their biological models, for example, by generating a large area of close contact and van der Waals (vdW) attachment forces. Bioinspired adhesives can also show a similarity to their biological models in appearance rather than function, where structural or material properties are transferred from the biological to the synthetic adhesive (e.g., micropatterning of the contact interface in tree-frog-inspired adhesives). The focus on specific geometric or material features of a biological model can lead to the reduction or even loss of functionality and performance (Farzaneh and Lindemann 2019).

The design of bioinspired fibrillar adhesives and analogies between these adhesives and their biological models have been addressed in various reviews (von Byern and Grunwald 2010; Kamperman et al. 2010; Jagota and Hui 2011). In contrast, reviews on ‘smooth’—primarily tree-frog-inspired—adhesives are scarce and mostly focused on the role of surface geometry and material properties to attachment (unless specified otherwise, we refer with ‘attachment’ to the combination of adhesive and frictional attachment forces; Barnes 2007; Chen et al. 2020; Meng et al. 2019; Zhang et al. 2019). In order to evaluate the functionality and attachment performance of synthetic ‘smooth’ adhesives, we review the functional analogy between these adhesives and the tree frog adhesive apparatus as their biological paradigm. We investigate which functional analogies have already been implemented in synthetic adhesives, and which hypotheses on the fundamental mechanisms of tree frog attachment are supported by experimental work on synthetic adhesives. Finally, we offer perspectives for the design of functionally analogous tree-frog-inspired adhesives.

## Principles of tree frog attachment

**Fig. 1:**
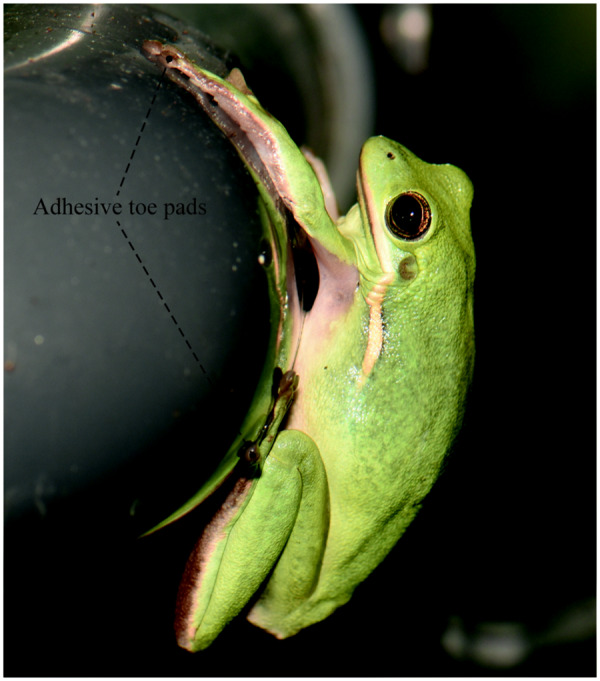
A tree frog (*Hyla cinerea*) clinging with its adhesive toe pads to a substrate.

Tree frogs possess adhesive pads at the tips of their toes (henceforth referred to as ‘frog pads’) for attachment in an arboreal habitat (Fig. 1). The morphology and functionality of these pads are subject to performance requirements such as the need to repeatedly attach and detach on various, often wet, substrates (Langowski et al. 2018a). Below, we review the mechanisms of tree frog attachment over a typical contact cycle (i.e., contact formation, attachment, and detachment) and discuss the morphological components of these mechanisms in frog pads.

### Contact formation

Attachment strength scales positively with the size of the contact area, and inversely with the distance between adhesive and substrate (Popov 2010). In tree frogs, the formation of close contact can be impeded by the roughness and liquid coverage of the various substrates encountered by these animals (Endlein et al. 2013a). Several mechanisms embodied in the toe pad structure and materials provide the pads with the ability to still form a sufficiently large area of close contact.

**Fig. 2:**
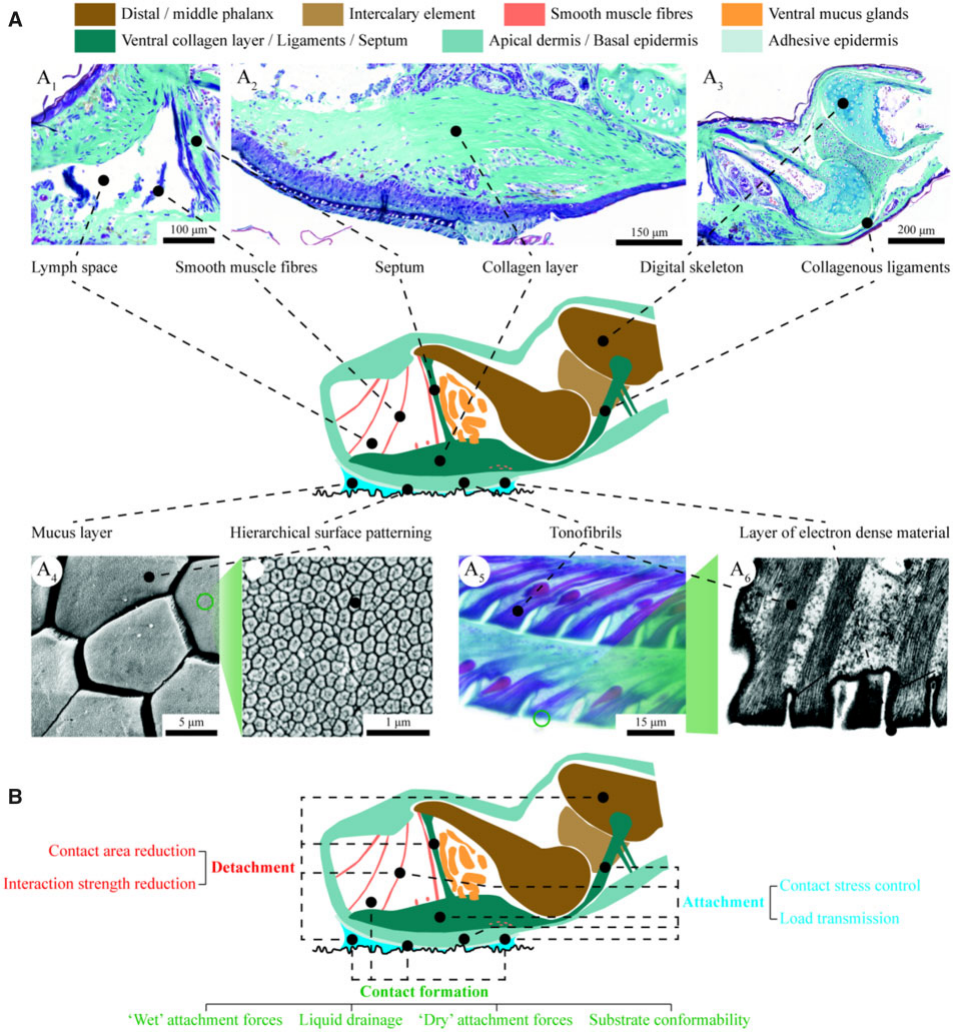
Schematic depiction of a tree frog’s toe pad in lateral view showing (**A**) structures and materials relevant to attachment (A_1–3_ lateral histographs, A_4_ ventral scanning electron micrographs, A_5_ lateral histograph, A_6_ lateral transmission electron micrograph), and (**B**) associated mechanisms of contact formation, attachment, and detachment (see main text for details). Micrographs modified with permission according to A_1–3,5_  Langowski et al. (2018b); A_4_  Federle et al. (2006); A_6_  Ernst (1973).

***Substrate conformability***: With an effective compressive elastic modulus E* of about 30 kPa (Scholz et al. 2009; Barnes et al. 2011), frog pads are soft on multiple scales, which facilitates conformation to nano- and microrough substrates (Crawford et al. 2016; Langowski et al. 2019a). Specifically, a hierarchical pillar-pattern on the ventral pad epidermis (Fig. 2A_4_; Noble and Jaeckle 1928; Ernst 1973; Green 1979) reduces the structural stiffness of the pad surface compared to a non-patterned adhesive, and individual nano- and micropillars presumably can fill substrate crevices of corresponding size levels to increase the effective contact area. The diameter of the micropillars scales inversely with species size (Smith et al. 2006). Next to the structure-based reduction of pad stiffness, frog pads comprise soft-material components such as lymph-filled spaces and a network of blood capillaries, which cushion the micropatterned epidermis (Fig. 2A_1_; Nakano and Saino 2016). A thin layer of relatively stiff electron dense material covers the pad surface (Fig. 2A_6_; Ernst 1973) and presumably protects the pad against mechanical wear (Langowski et al. 2018a).

***Liquid drainage***: A hierarchical pattern of pillar-like structures separated by channels as found on the pad surface has been shown theoretically (Persson 2007) and experimentally (Gupta and Fréchette 2012) to drain interstitial liquids at (sub-)micrometric pad-substrate gap widths. Frog pads typically are curved convexly (Barnes et al. 2011), which may ease the expulsion of liquids from the periphery of the pad-substrate gap (Kaveh et al. 2014; Langowski et al. 2018a). Furthermore, the mucus covering the pad surface has a low viscosity (Federle et al. 2006), which reduces viscous forces and thereby likely alleviates drainage of the skin-inherent mucus layer. A similar effect may be caused by the low surface tension of the mucus (Drotlef et al. 2013; Langowski et al. 2019b), which presumably leads to strong wetting, the distribution of a given mucus volume over a large surface area, and thus to a reduced height of the skin-inherent mucus layer.

***‘Dry’ attachment forces***: Substrate conformability and liquid drainage facilitate the formation of areas of ‘dry’ contact with gap widths smaller than ca. 10 nm (Federle et al. 2006), which is potentially sufficiently close for the generation of vdW forces (Federle et al. 2006; Langowski et al. 2018a). Moreover, the thin layer of electron dense material covering the pad surface possibly amplifies the strength of vdW interactions (Langowski et al. 2018a).

***‘Wet’ attachment forces***: The interstitial mucus layer has been hypothesized to facilitate capillary and hydrodynamic adhesion—so called ‘wet adhesion’ (Emerson and Diehl 1980; Hanna and Barnes 1991)—which may increase the effective contact area on rough substrates (Barnes 1999).

### Attachment

Once a large area of close contact has been established, the generated contact needs to be maintained. This requires sufficient mechanical strength of the soft pad to withstand external loads such as body weight and inertial forces (Bijma et al. 2016). Unwanted detachment, which occurs due to local contact stress concentrations exceeding the maximal attachment strength (Bacca et al. 2016), can be avoided by the control of the spatial distribution (and total amount) of contact stresses (Gao and Yao 2004).

***Load transmission***: The micropatterned skin of a frog pad is not a self-contained adhesive system but forms an adhesive ‘anchor point’ for the skeletomuscular system. The mechanical link between the pad surface and the rest of the body is formed by anisotropic networks of keratinous tonofibrils (Fig. 2A_5,6_; Ernst 1973; Nakano and Saino 2016), which run from the contact surface to the basal membrane of the epidermis, and a layer of collagen fibers (Fig. 2A_2_; Langowski et al. 2018b), which connect the basal membrane of the epidermis via collagenous ligaments with the digital skeleton (Fig. 2A_3_). These networks are strong and stiff in tensile loading, and presumably mechanically strengthen the pad (Langowski et al. 2018b).

***Contact stress control***: The overall architecture of an adhesive organ determines the spatial distribution of contact stresses (Gorb et al. 2007) and can facilitate a uniform stress distribution (Gao and Yao 2004), which would strengthen attachment. Tree frogs may actively modulate the contraction of pad-intrinsic smooth muscle fibers (Fig. 2A_1_) to create a favorable contact stress distribution upon external disturbances and, conversely, enable detachment (Langowski et al. 2018b).

### Detachment

At the end of the contact cycle, a tree frog needs to reduce adhesion, so it detaches its pads from the substrate. Generally, animals switch from attached to detached state by reducing contact area and contact strength (Federle and Labonte 2019).

***Contact area reduction***: The adhesion of frog pads scales positively with the applied shear load (Federle and Labonte 2019). This scaling may be partially explained by a positive proportionality between shear load and contact area caused by the structural properties of the fiber-reinforced epidermis of frog pads, as observed in the fiber-reinforced adhesive pads of stick insects (Dirks et al. 2012). Next to this geometric effect linking shear load with contact area, a release of elastic energy stored in the fiber-networks found in frog pads may facilitate contact area reduction, as described for various hairy attachment systems (Federle and Labonte 2019). Finally, it has been hypothesized that tree frogs use their pad-intrinsic musculature to modify pad shape and thus the size of the available contact area (Langowski et al. 2018b).

***Contact strength reduction***: According to the theory of tape peeling, attachment strength scales inversely with the angle between the substrate surface and the load vector acting on an adhesive (Kendall 1975). Tree frogs (Barnes et al. 2008; Endlein et al. 2013b) and other animals (Federle and Labonte 2019) presumably use peeling to control attachment strength. Additionally to the collagen layer, a dorsal-ventral septum connects the adhesive pad surface to the digital phalanx (Fig. 2A_1_). Loading the adhesive surface via either one of these structures arguably leads to different contact stress distributions across the ventral pad surface, causing firm attachment when loading the collagen layer, and detachment when loading the septum (Langowski et al. 2018b). Attachment strength may also depend on the thickness of the interstitial liquid film. Liquid drainage during pad sliding has been shown to strengthen attachment in insects (Federle and Labonte 2019). Conversely, tree frogs may actively ‘flood’ the pad-substrate gap with mucus to reduce attachment strength, or add surfactants to the mucus to control attachment strength (Langowski et al. 2019b).

## Existing tree-frog-inspired adhesives

**Fig. 3:**
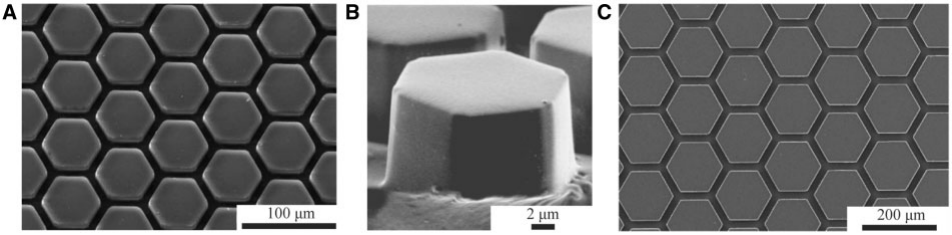
Examples of tree-frog-inspired ‘smooth’ adhesives. (**A**) Hexagonal PDMS surface pattern for friction under dry conditions (Murarash et al. 2011), (**B**) Hexagonally micropatterned PDMS surface for capillary adhesion (Drotlef et al. 2013), (**C**) Hexagonal PDMS surface for friction under wet conditions. See also Table 1. All panels reproduced with permission.

Many researchers have studied the versatile attachment of tree frogs to design biomimetic adhesives (Barnes 1999; Barnes et al. 2002). Most adhesives resulting from these studies (Table 1) mimic the epidermal surface of frog pads (Fig. 3). These adhesives demonstrate structural rather than functional analogy, as shown by the lack of hierarchal micropatterning and the uniform properties of the used materials.

**Table 1:** Overview of studies on tree-frog-inspired adhesives bearing hexagonal (H) or cylindrical (C) pillars tested in a dry (D) or wet (W) environment, in chronological order

Reference	Pillar design			Measurement		*d*_m_	*h*_m_	*w*_m_
	Shape	Material	Manufacturing method	Adhesion	Friction	(µm)	(µm)	(µm)
Varenberg and Gorb (2009)	H	PVS	MO-1(Steel)	—	D, W	≈9–82	1	—
Murarash et al. (2011)	H	PDMS	MO-1(SU-8)	—	D	50	10–50	—
Drotlef et al. (2013)	H, C	PDMS	MO-1(SU-8), MO-2(PDMS)	D, W	D, W	7–15	5–20	4–8
Tsipenyuk and Varenberg (2014)	H	PVS	MO-1(SU-8), 3D(Glass)	—	W	50–610	25–510	—
Dhong and Fréchette (2015)	C	SU-8	PL	W	—	10	10	3–10
Iturri et al. (2015)	H^a^	PDMS	MO-2(PDMS)	—	D, W	15	5–20	3
Chen et al. (2015)	H^a^	PDMS	MO-1(SU-8)	—	W	140	30	20
Li et al. (2015)	H-C^b^	PDMS	PL/WE/MO-1	—	W	≈200	12	≈7
Zhang et al. (2016)	H	PDMS	—	—	W	120	35	20
Ko et al. (2017)	H	PDMS	MO-1(SU-8)	—	D, W	25	10–40	2.5–50
Xue et al. (2017)	C^c^	PDMS (PS)	MO-3/SM	D	D	17	5	3
Li et al. (2018a)	H	PDMS	MO-1(AZ P4620)	W	—	≈20	8.5	≈20
Xie et al. (2018)	H	CPUE	MO-2(PDMS)	D, W	—	≈40–100	45–105	14–214
Gong et al. (2018)	H, C	PDMS	MO-1	D, W	D, W	100–200	≈20–350	5
Chen et al. (2018)	H	Bronze	MI	—	W	10000	1000	1000
Feng et al. (2019)	H, C	SR, TPU	MO	—	D	300	50	30
Liu et al. (2020)	C^d^	PDMS	MO-1(SU-8)	W	—	20	5	20
				
	**Biological model—tree frog**							
Langowski et al. (2018a)	Epidermal cells					≈10	≈10	≈1
Federle et al. (2006)	Nanopillars					0.31	0.22	0.02

a Elongated hexagonal pillar outlines.

b Hierarchical micropattern.

c Fiber-reinforced micropillars.

d Nanodimples.

Manufacturing methods: MI, milling; MO-*N*(X), molding in *N* steps with final mold material X; PL, photolithographic processing; SM, stamp molding; WE, wet etching; 3D(X), 3D-printing on substrate material X. *d*_m_, diagonal pillar diameter; *h*_m_, pillar height; *w*_m_, channel width; CPUE, polyurethane elastomer; PS, polystyrene; PVS, polyvinylsiloxane; SR, silicone rubber; SU-8, epoxy based photoresist; TPU, thermoplastic polyurethane elastomer.

The comparison of attachment performance between the different tree-frog-inspired adhesives is challenging due to variations in micropillar dimensions and the methods used to measure attachment performance. Current tree-frog-inspired adhesives bear pillar arrays with pillar sizes of 10–500 µm (Table 1), that is, pillars that are generally larger than the pillar-like structures found on frog pads. This size difference contradicts the inverse scaling of pillar size with the size of the adhesive (i.e., load) observed in various bioadhesive systems (Arzt et al. 2003): the typically larger and heavier synthetic adhesives would require even smaller pillars than those found on frog pads to reach a similar adhesive performance as the biological system. Also, the role of hierarchical surface patterning, as observed in frog pads, has been barely studied (see Li et al. 2015; Liu et al. 2020 for exceptions).

Most existing synthetic adhesives have been made from polydimethylsiloxane (PDMS; Table 1) by positive or negative molding. PDMS is hydrophobic (Xie et al. 2018), whereas frog pads are hydrophilic (Drotlef et al. 2013). Moreover, PDMS is—with an elastic modulus in the order of 2 MPa (Xue et al. 2017)—about 100 times stiffer than the bulk material of the frog pads (Langowski et al. 2018a).

Compared to non-patterned adhesives, adding micropillars tends to increase adhesion (Li et al. 2018a) and friction on various hydrophilic substrates under wet conditions (Varenberg and Gorb 2009; Tsipenyuk and Varenberg 2014; Iturri et al. 2015; Li et al. 2015). In contrast, the presence of micropillars drastically reduces friction on dry substrates (Varenberg and Gorb 2009; Iturri et al. 2015). These effects suggest a benefit of low aspect-ratio pillars for attachment under wet rather than dry conditions. However, none of the hypotheses put forward in the literature to explain the optimal geometry of tree-frog-inspired micropatterning (e.g., an optimal ratio of pillar surface area to channel volume; Varenberg and Gorb 2009; Tsipenyuk and Varenberg 2014; Iturri et al. 2015; Ko et al. 2017; Kim et al. 2019) quantitatively predicts the effects of variations in shape and dimensions of the micropatterning on the performance of tree-frog-inspired adhesives, or considers contributions of the nanopatterning. Most previous studies only report effects of surface patterning (and of variations of size and geometry thereof) on attachment performance, and empirical evidence on the underlying mechanisms is often missing. Below, we attempt to extract such evidence from the findings on existing tree-frog-inspired adhesives and discuss functional analogies between these synthetic adhesives and frog pads.

### Contact formation

Our synthesis of the findings of past studies highlights that substrate conformability is an important mechanism in the attachment of tree-frog-inspired adhesives, and likely also in their biological models. Both a reduced material stiffness (Li et al. 2018a) and the addition of hierarchical layers of surface patterning (i.e., a reduced structural stiffness; Li et al. 2015) increase adhesion, which may be explained by close conformation to the substrate.

Adding a pattern of micropillars separated by a network of interconnected microchannels effectively enhances the gap width between adhesive and substrate separated by a liquid and—according to hydrodynamic theory—reduces the viscous resistance against flow of that liquid, which is caused by a change of the distance between adhesive and substrate (Pilkington et al. 2016). Thus, tree-frog-inspired micropatterning reduces hydrodynamic repulsion and adhesion between adhesive and substrate (Gupta and Fréchette 2012; Dhong and Fréchette 2015), which in turn allows close contact formation, particularly at gap widths <1 µm. We hypothesize that this drainage of interstitial liquids explains the stronger adhesion and friction (Drotlef et al. 2013; Chen et al. 2015) of submerged tree-frog-inspired adhesives compared to non-patterned adhesives. Chen et al. (2015) showed that drainage efficiency, approximated by the generated friction, is anisotropic and scales positively with the relative channel length in the direction of shear loading. The aforementioned mechanisms presumably facilitate a large area of close contact, which is a prerequisite to generate ‘dry’ vdW forces, even if an interstitial liquid layer is present (Langowski et al. 2018a). In fact, Dhong and Fréchette (2015) suggested that micropatterned adhesives can reach boundary contact, which is potentially close enough for the generation of vdW forces (Langowski et al. 2018a). Micropatterning also impedes the propagation of a crack between adhesive and substrate, and thus strengthens attachment (Ghatak et al. 2004). This so-called ‘crack arresting’ can be indicative of the action of vdW forces, and has been suggested to occur in frog-inspired adhesives both under wet and dry conditions (Iturri et al. 2015; Kim et al. 2019). Furthermore, the characteristics of force-distance curves measured on micropatterned adhesives under wet conditions suggest the action of vdW forces; these forces can contribute significantly (>50%) to the total adhesion (Drotlef et al. 2013; Li et al. 2018a). Finally, adhesion and friction scale with the effective contact area (Murarash et al. 2011; Gong et al. 2018), which may imply the action of contact-area-dependent vdW forces. Overall, we show that various experimental observations support the action of ‘dry’ vdW forces in the attachment of tree-frog-inspired adhesives under wet conditions, and possibly also in tree frogs.

It has been suggested that tree-frog-inspired adhesives can also generate ‘wet’ attachment forces (Drotlef et al. 2013; Li et al. 2018a) via capillary and hydrodynamic effects. Micropatterns of pillars separated by channels can reduce the range of the generated capillary forces by sucking liquid into the channels and thus diminishing the volume of the liquid layer (Drotlef et al. 2013). Accordingly, micropatterning may be even detrimental for the attachment of micropatterned adhesives using wet adhesion.

### Attachment

The surface of an adhesive determines the mechanisms of contact formation and hence ordains attachment strength. Strong and lasting attachment, however, is co-determined by internal structures. For example, a PDMS adhesive with fiber-reinforced pillars creates stronger attachment than one with homogenous pillars (Xue et al. 2017). Tree-frog-inspired fiber-reinforcement has been shown to facilitate a shift of the maximum interfacial contact stress from the edge to the central region of the pillar contact area and reduces the maximum contact stress (Xue et al. 2017), which both hinder crack initiation. Similarly, fiber-reinforcement in gecko-inspired adhesives strengthens attachment via so-called shear-stiffening (Bartlett et al. 2012b). It remains for future work to elucidate which load-transmission-related mechanisms are dominant in tree-frog-inspired adhesives and their biological models. We expect that load transmission occurs in frog pads differently to existing synthetic adhesives because the epidermal cells and the tonofilaments therein are skewed (Ernst 1973; Langowski et al. 2018b).

### Detachment

Peeling is a general mode of detachment in tree-frog-inspired adhesives (Drotlef et al. 2013; Dhong and Fréchette 2015). However, only few detailed analyses exist on the reduction mechanisms of contact area and interaction strength. Drainage due to micropatterning facilitates contact formation and the release of contact (Dhong and Fréchette 2015). For synthetic adhesives under wet conditions, attachment strength is sensitive to the interstitial liquid volume; whereas the addition of small amounts (0.1–0.3 µL) of water strengthens wet adhesion, a further increase in liquid volume reduces adhesion and friction (Drotlef et al. 2013; Zhang et al. 2016; Gong et al. 2018). Reduced adhesion due to wetting has also been observed in tree frogs (Meng et al. 2019), and we propose that the control of the volume (and thus thickness) of the liquid bridge is an important detachment mechanism both in bioinspired and biological adhesives. Besides volume, also the chemical composition of the liquid plays a role in detachment. Low concentrations (<0.05%) of a surfactant in an interstitial water film stabilize the liquid layer and hence reduce the contribution of vdW forces to the attachment of tree-frog-inspired adhesives (Li et al. 2018b). Considering the presence of surfactants in the mucus of tree frogs, modifications of the mucus chemistry may play an important role also in the detachment of the biological model (Langowski et al. 2019b). In summary, we show that most findings on synthetic adhesives support a role of the liquid layer (and modifications thereof) in detachment.

## Perspectives for the design of tree-frog-inspired adhesives

We compiled evidence for two attachment-related mechanisms in synthetic adhesives that are enabled by tree-frog-inspired micropatterning compared to non-patterned adhesives: (1) Hexagonal surface patterning facilitates—in particular on hydrophilic substrates—drainage, which helps to remove interstitial liquids, form close contact, and generate vdW forces. (2) Micropatterning may hinder crack propagation by crack arresting, hence enhancing attachment. More importantly, we show that surface patterning alone is not sufficient to reach functional analogy with tree frogs. Below we offer some perspectives on steps that may bring us closer to this goal.

### Novel design concepts

**Fig. 4:**
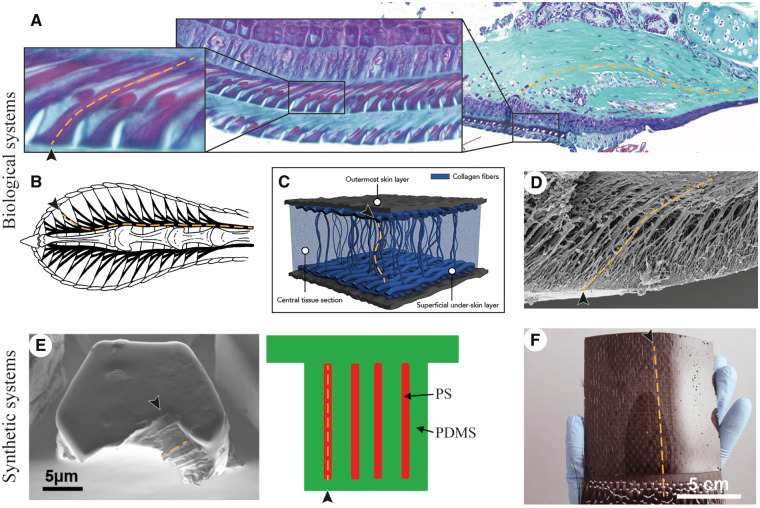
Biological (**A**–**D**) and synthetic (**E**–**F**) adhesives with internal fiber-reinforcements connecting the contact surface (arrowhead) with deeper regions of the respective system (yellow dashed line). (A) Keratinous tonofilaments (left) and collagenous fibers (right) in the toe pad of the tree frog *Hyla cinerea* (modified after Langowski et al. 2018b). (B) Network of tendons in the toe pad of the gecko *Gekko gecko* (modified after Russell 1975). (C) Collagen fibers in the suction disc of the remora fish (modified after Su et al. 2020). (D) Cuticular fibrils in the adhesive pad of the stick insect *Carausius morosus* (modified after Dirks et al. 2012). (E) Synthetic micropillars with embedded nanofibers in top view (left) and in schematic lateral section (right; modified after Xue et al. 2017). (F) Smooth polymer surface with underlying carbon fiber fabric (Bartlett et al. 2012b). All panels reproduced with permission.

The implementation of internal fiber-reinforcements inspired by the keratinous and collagenous structures in the adhesive pads of tree frogs (see ‘Contact formation’ section) and other animals could help to create mechanically stronger adhesives (Fig. 4). Many existing tree-frog-inspired adhesives consist of a micropillar pattern that is connected via a soft homogeneous base layer to a stiff support (e.g., Drotlef et al. 2013). Such mounting can reduce the attachment performance of the adhesive by altering its mechanical properties and can lead to damage of the base layer due to local overloading (Xue et al. 2017). We propose to replace the stiff support with an internal fiber-network inspired by the ﬁbrous structures in frog pads for protection against internal cohesive failure.

Furthermore, fiber-reinforcements cause anisotropic material behavior. In compression, soft fiber-reinforced adhesives can conform closely to rough substrates, and thus form a large contact area for the generation of vdW forces. During tensile loading, fibrous structures stiffen the pad and may optimize the spatial distribution of contact stresses for strong attachment, as indicated by Xue et al. (2017). The benefit of such an anisotropic stiffness in compression and tension has been formulated in a generalized scaling law ($F\propto \sqrt{AK}$; with attachment force capacity *F*, contact area *A*, and stiffness *K* of the adhesive; Bartlett et al. 2012b), which explains the scaling of *F* of various biological and technical systems for a range of variations of *A* and *K* by factors of up to 10^10^ (Bartlett et al. 2012a, 2012b; Gilman et al. 2015). Although the underlying physical mechanism is still under debate (Mojdehi et al. 2017), the presence of fibrous elements in the surface region of the adhesive organs of geckos (Autumn and Peattie 2002), insects (Gorb and Beutel 2001), remora suckerfish (Su et al. 2020), and tree frogs emphasizes the potential of fiber-reinforcement for the design of bioinspired adhesives, particularly for applications where strong attachment is required (e.g., soft heavy-duty grippers or fast moving robots). Alternatively, conformable yet mechanically resistant micropatterned adhesives may be realized by the combination of a relatively stiff superficial layer with a relatively soft underlying bulk material.

Next to providing mechanical strength and strong attachment, fiber-reinforcement also facilitates repeated attachment by preventing lateral bending and clustering of pillars, as well as the resulting permanent decrease in attachment performance (Bae et al. 2013; Xue et al. 2017). Various assemblies of force-transmitting structures could get implemented in future biomimetic adhesives to modulate attachment strength, analogously to the different pathways of force-transmission in frog pads (i.e., collagen layer and dorsal-ventral septum).

Attachment control is presumably also facilitated by the smooth muscle fiber bundles found in frog pads (see ‘Attachment’ section). To our knowledge, the hypothesized functionality of these structures has not yet been transferred into technical systems. In existing actuated micropatterned adhesives, switchable adhesion is mostly achieved by modulations of the pillar geometry using external stimuli such as temperature (Reddy et al. 2007; Cui et al. 2012) or a magnetic field (Northen et al. 2008; Drotlef et al. 2014). Furthermore, switchable adhesion by modification of the overall contact surface topology is accomplished using pneumatic (Nadermann et al. 2010) and electric (Shivapooja et al. 2013) stimuli. Other solutions utilize a hysteresis in the buckling of micropillars (Paretkar et al. 2011; Purtov et al. 2015) to switch between adhesive and non-adhesive state. All these adhesives can switch from an ‘attached’ to a ‘detached’ state rather than gradually transitioning from one state to the other. Moreover, most existing actuated adhesives rely on specific modifications of the topology of the adhesive surface. The implementation of tree-frog-inspired contractile components in the base layer of a micropatterned adhesive could allow the gradual control of attachment via modification of the adhesive’s stiffness and contact area while maintaining freedom in the design of the surface pattern.

We expect that reverse engineering of frog pads as proposed above will also generate new knowledge on the attachment of tree frogs itself. For example, mechanistic insight on fiber-reinforced synthetic adhesives may help to understand the mechanical connection between the epidermal tonofilaments and the surrounding cellular matrix. A combination of numerical (e.g., fluid-structure-interaction simulations) and experimental approaches such as measuring the effects of systematic variations of the micropattern geometry on the attachment performance may help to identify key functions (e.g., stiffness reduction, liquid drainage, force transmission) of the micropatterning of frog pads. Also, the role of interstitial liquid in attachment and detachment can be studied more easily in synthetic adhesives than in biological ones. Is tree frog mucus really needed for attachment, or does it fulfill other functions? Finally, we are not aware of studies on the sensing of attachment in tree-frog-inspired adhesives or their biological paradigm. What attachment modalities do tree frogs sense, and where should one embed what type of sensor in synthetic adhesives for attachment control?

### Tackling technical challenges

The design of functionally analogous tree-frog-inspired adhesives requires not only a better understanding of the fundamentals of tree frog attachment, but also the solution of challenges in the manufacturing of synthetic adhesives. Sufficiently large samples with microscopic surface patterning, as required to produce tree-frog-inspired micropatterned adhesives, are fabricated mostly with parallel methods such as molding (Table 1). With molding methods, however, complex 3D architectures such as structures with internal spaces cannot be made, except by means of complex post-processing (Assenbergh et al. 2018). Complex architectures can be fabricated with serial methods (e.g., lithography and photopolymerization). However, the throughput of serial methods is typically limited to a few micrometers per second. To illustrate, the fabrication of a patterned adhesive with a surface area of 1 cm^2^ and with 20 nm large features might take as long as 24 h (Gates et al. 2005). Larger structures can be fabricated with additive manufacturing (3D printing), but this goes at the expense of spatial resolution. Moreover, it is technically challenging to fabricate structures made of multiple materials (e.g., a soft adhesive surface with stiff fibers for reinforcement). The use of multiple materials in combination with molding has rarely been demonstrated for the fabrication of micropatterns (see Bae et al. 2013; Xue et al. 2017 for exceptions). Multimaterial 3D printing is possible, but again at the cost of spatial resolution. While some of the aforementioned limitations are intrinsic to the method used (e.g., the incompatibility of molding techniques with complex 3D architectures), other limitations could in principle be conquered with technical progress. For example, currently 3D printing cannot be used to fabricate micro- and nanofeatures, but the pixel volume of 3D printing is decreasing with time.

Alternative design concepts might be considered to develop adhesives with properties similar to those of their biological models. For example, while some fabrication techniques prohibit the use of materials with a wide range of material stiffness, stiffness variation could be achieved by means of structural modifications instead (e.g., overall geometry, porosity, and wall thickness; see e.g., Ko et al. 2017). Finally, novel techniques could get used in combination with conventional ones to create bioinspired adhesives in a bottom-up process from single building blocks. For instance, a 3D-printed scaffold could be used to grow biological material in a biosynthetic approach. Also, developments in closely related fields such as soft robotics—for example, the development of novel embedded sensors and actuation technologies (Polygerinos et al. 2017; Jiang et al. 2019)—may help to advance the design of tree-frog-inspired adhesives that are functionally analogous to their biological models.

### Concluding remarks

Overall, tree frogs are fascinating models for the design of ‘smooth’ adhesives that function under challenging conditions, and many application fields may benefit from tree-frog-inspired technologies. Due to their strong attachment to a large range of substrates, varying in surface energy (personal observation by J.K.A. Langowski) and roughness (Langowski et al. 2019a), under wet conditions, tree-frog-inspired adhesives may be applied in fields such as microfluidics (Dhong and Fréchette 2015), medical equipment (Varenberg and Gorb 2009; Feng et al. 2019), wearables (Drotlef et al. 2013; Chen et al. 2020), or personal hygiene products (Tsipenyuk and Varenberg 2014). Furthermore, fiber-reinforced adhesives could be used in high-load-applications such as climbing robots (Meng et al. 2019), car tires (Barnes 2007), and soft robotic grippers (Nguyen and Ho 2018, 2019).

Importantly, the attachment apparatus of tree frogs is a complex system of interconnected functional components. The transfer of individual components into tree-frog-inspired adhesives may be sufficient for specific applications (e.g., the implementation of micropatterned surface for drainage of interstitial liquids) but likely leads to a reduced performance compared to the biological model. Therefore, future work should focus on an integrative approach that takes into account the different functional components of frog pads. Furthermore, the design of future frog-inspired adhesives should target a better understanding of the fundamental mechanisms of tree frog attachment as a solid foundation for the improvement of synthetic adhesives. As this review highlights the importance of ‘dry’ contact in the ‘wet’ adhesive toe pads of tree frogs, future work on tree frog attachment may uncover further conceptual similarities between ‘dry’ and ‘wet’ adhesive systems (as suggested by Wang et al. 2017), and we expect that both subfields of bioadhesion could benefit from such exchange of concepts.

## Funding

This work is part of the research programme “Secure and gentle grip of delicate biological tissues” with project number 13353, which is financed by the Netherlands Organisation for Scientific Research (NWO). Further funding was received within the Soft Robotics Consortium with project number 4TU-UIT-335 (internal: 4162504200), which is financed by the 4TU.Federation.

## Authors’ contributions

J.K.A.L. reviewed the literature, designed the figures, and wrote the first manuscript draft. All authors commented on the outline, revised the manuscript, and approved the final version of the manuscript.

## Notes

From the symposium “Form, Structure and Function: How Plants vs. Animals Solve Physical Problems” presented at the annual meeting of the Society for Integrative and Comparative Biology January 3–7, 2020 at Austin, Texas.
